# Dimethyl 11,13-dimethyl-16-[1,2-bis­(methoxy­carbon­yl)ethen­yl]-12-oxo-16,17-dioxa-18-aza­hexa­cyclo­[7.5.1.1^1,4^.1^6,9^.1^10,14^.0^5,15^]octa­deca-2,7-diene-2,3-dicarboxyl­ate

**DOI:** 10.1107/S1600536809050600

**Published:** 2009-11-28

**Authors:** Atash V. Gurbanov, Eugeniya V. Nikitina, Elena A. Sorokina, Fedor I. Zubkov, Victor N. Khrustalev

**Affiliations:** a Department of Chemistry, Baku State University, Z. Khalilov St. 23, Baku AZ-1148, Azerbaijan; b Organic Chemistry Department, Russian Peoples Friendship University, Miklukho-Maklaya St. 6, Moscow 117198, Russian Federation; c X-Ray Structural Centre, A. N. Nesmeyanov Institute of Organoelement Compounds, Russian Academy of Sciences, 28 Vavilov St., B–334, Moscow 119991, Russian Federation

## Abstract

The title compound, C_27_H_29_NO_11_, is a product of the tandem ‘domino’ Diels–Alder reaction. The mol­ecule comprises a fused hexa­cyclic system containing four five-membered rings (two dihydro­furan and two tetra­hydro­furan) in the usual envelope conformations and two six-membered rings (tetra­hydro­pyridinone and piperidine) adopting slightly flattened boat and chair conformations, respectively. The dispositions of the carboxyl­ate substituents relative to each other are determined by both steric reasons and inter­molecular C—H⋯O hydrogen bonding and attractive anti­parallel C=O⋯C=O inter­actions [C⋯O = 2.995 (2) Å].

## Related literature

For the tandem ‘domino’ Diels–Alder reaction, see: Wasserman & Kitzing (1969 ▶); Winkler (1996 ▶); Padwa & Bur (2007 ▶). For related compounds, see: Lautens & Fillion (1996 ▶, 1997 ▶); Domingo *et al.*, (2000 ▶).
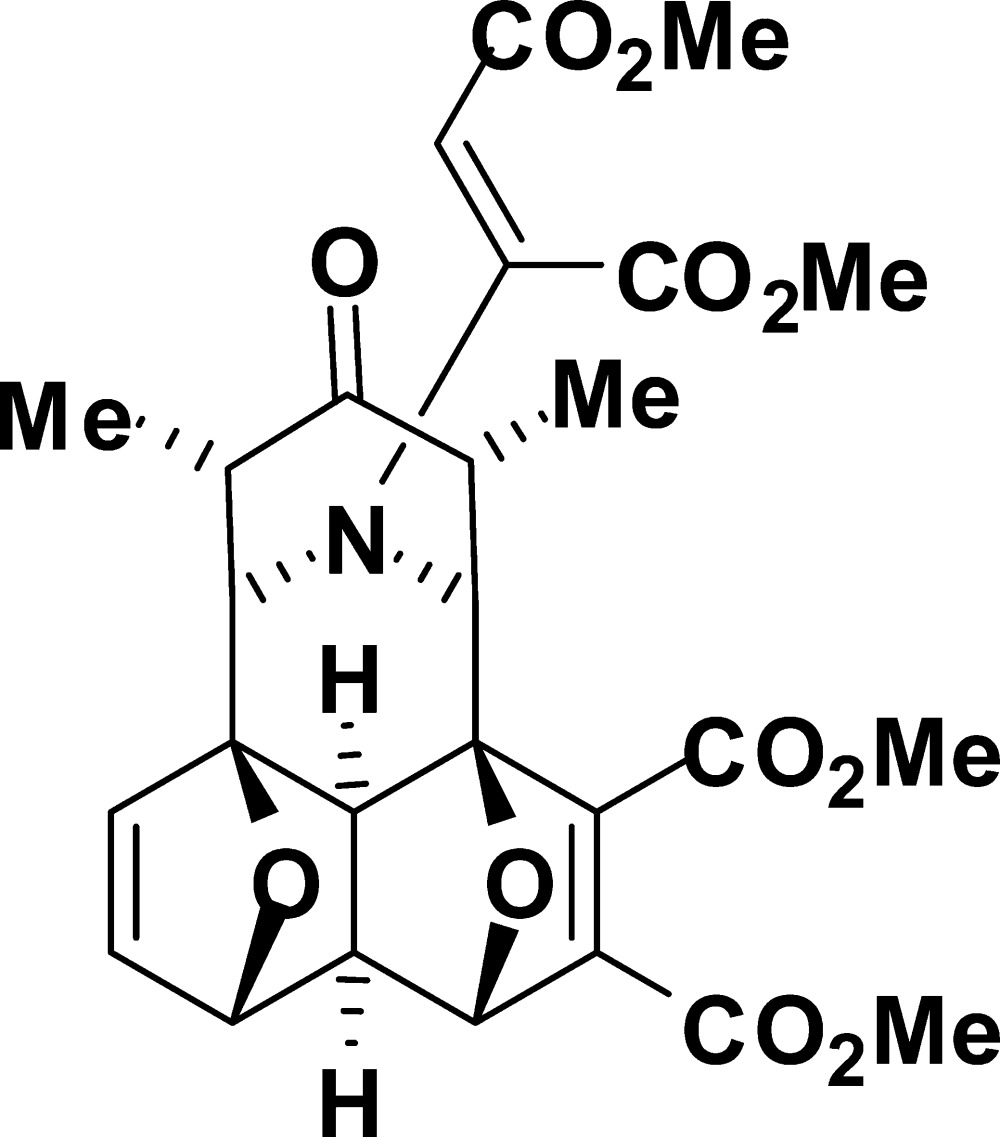


## Experimental

### 

#### Crystal data

C_27_H_29_NO_11_
*M* *_r_* = 543.51
Monoclinic, 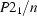

*a* = 16.1430 (7) Å
*b* = 9.1365 (4) Å
*c* = 16.9658 (7) Å
β = 95.019 (1)°
*V* = 2492.70 (18) Å^3^
*Z* = 4
Mo *K*α radiation
μ = 0.11 mm^−1^
*T* = 120 K
0.30 × 0.25 × 0.20 mm

#### Data collection

Bruker SMART 1K CCD diffractometer
Absorption correction: multi-scan (*SADABS*; Sheldrick, 1998 ▶) *T*_min_ = 0.966, *T*_max_ = 0.975
27638 measured reflections
7186 independent reflections
5342 reflections with *I* > 2σ(*I*)
*R*_int_ = 0.041

#### Refinement

*R*[*F* ^2^ > 2σ(*F* ^2^)] = 0.054
*wR*(*F* ^2^) = 0.151
*S* = 1.00
7186 reflections
358 parameters
H-atom parameters constrained
Δρ_max_ = 0.48 e Å^−3^
Δρ_min_ = −0.27 e Å^−3^

### 

Data collection: *SMART* (Bruker, 1998 ▶); cell refinement: *SAINT-Plus* (Bruker, 1998 ▶); data reduction: *SAINT-Plus*; program(s) used to solve structure: *SHELXTL* (Sheldrick, 2008 ▶); program(s) used to refine structure: *SHELXTL*; molecular graphics: *SHELXTL*; software used to prepare material for publication: *SHELXTL*.

## Supplementary Material

Crystal structure: contains datablocks global, I. DOI: 10.1107/S1600536809050600/rk2182sup1.cif

Structure factors: contains datablocks I. DOI: 10.1107/S1600536809050600/rk2182Isup2.hkl

Additional supplementary materials:  crystallographic information; 3D view; checkCIF report

## Figures and Tables

**Table 1:** Hydrogen-bond geometry (Å, °)

*D*—H⋯*A*	*D*—H	H⋯*A*	*D*⋯*A*	*D*—H⋯*A*
C19—H19*C*⋯O8^i^	0.98	2.70	3.011 (2)	99

Symmetry code: (i) 
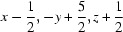
.

**Fig. 1.**
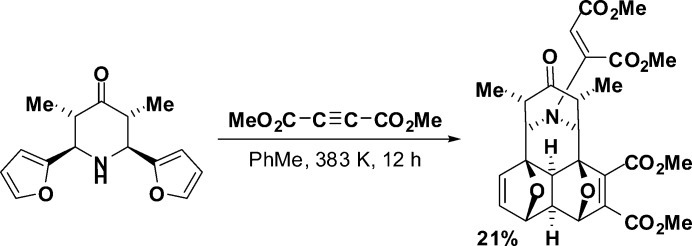
Construction of bridged polyheterocyclic system using the tandem "domino" Diels–Alder reaction.

**Fig. 2.**
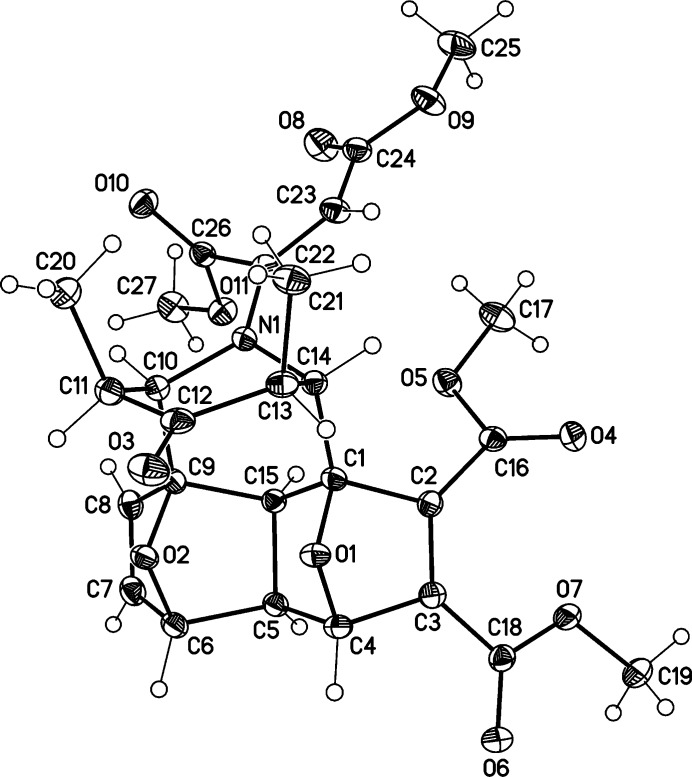
Molecular structure of I with the atom numbering scheme. Displacement ellipsoids are shown at the 50% probability level. H atoms are presented as a small spheres of arbitrary radius.

## supplementary crystallographic information

### Comment

The tandem "domino" Diels–Alder reaction consists of two consecutive Diels–Alder cycloadditions between two dienes and an acetylenic dienophile acting as a bisdienophile. To the best of our knowledge, only a few papers on tandem "domino" Diels–Alder reaction for furan species (when furan derivatives are used as the diene components) have been published (Lautens & Fillion, 1996, 1997; Domingo *et al.*, 2000). Meanwhile, the tandem "domino" Diels–Alder reaction gives rise to the rapid construction of bridged polyoxacyclic systems (Wasserman & Kitzing, 1969; Winkler, 1996; Padwa & Bur, 2007). This work demonstrates the stereoselectivity (*exo*–*exo*–adduct) of the reaction of 2,6–difurylpiperidinone with dimethyl acetylene dicarboxylate (*DMAD*). The reaction passes through initial Michael addition of *DMAD* molecule to the nitrogen atom of piperidone with subsequent (4 + 2) cycloaddition of another *DMAD* molecule by the furan ring. The intramolecular "domino" Diels–Alder reaction within the generated adduct completes the process on the last stage (Fig. 1).

The molecule of title compound, I, comprises a fused hexacyclic system containing four five–membered rings (two dihydrofuran and two tetrahydrofuran) and two six–membered rings (tetrahydropyridinone and piperidine) (Fig. 2). All four five–membered rings of the tetracyclic fragment have usual *envelope* conformations, and the six–membered rings adopt the slightly flattened *boat* and *chair* conformations, respectively. The nitrogen N1 atom has a trigonal–planar geometry (sum of the bond angles is 359.68 (12)°). The dihedral angle between the planes of the tetrahydropyridinone and piperidine rings is 66.48 (8)°. The carboxylate ligand at the C3 carbon atom lies practically in the plane of the O1C1C2C3C4 dihydrofuran ring (the C2—C3—C18—O7 torsion angle is -8.1 (2)°), while that at the C2 carbon atom is turned out of this plane (the C3—C2—C16═O4 torsion angle is -52.2 (3)°). Such disposion of the carboxylate substituents is determined by both steric reasons and intermolecular C19—H19C···O8^i^ hydrogen bond [C19···O8^i^ = 3.011 (2)Å, H19C···O8^i^ = 2.70Å, C19—H19C···O8^i^ = 99°] and attractive antiparallel C16═O4···C16^ii^═O4^ii^ interactions [C16···O4^ii^ = 2.995 (2)Å]. The methyl substituents at the C11 and C13 carbon atoms occupy the sterically unfavourable axial positions, which can be explained by the direction of the intramolecular "domino" Diels–Alder reaction. Symmetry codes: (i) -1/2+*x*, 2.5-*y*, 1/2+*z*; (ii) 1-*x*, 2-*y*, -*z*.

The molecules I are diastereomers and possess ten asymmetric centers at the C1, C4, C5, C6, C9, C10, C11, C13, C14 and C15 carbon atoms. The crystal of I is racemate and consists of enantiomeric pairs with the relative configuration of the centers *rac*–1*R**,4*S**,5*R**,\ 6*S**,9*R**,10*R**,11*S**,\ 13*R**,14*S**,15*S**.

### Experimental

The solution of (2*R**,3*S**,5*R**,6*S**)-2,6-di(2-furyl)-3,5-dimethylpiperidin-4-one (1.0 g, 3.86 mmol) and *DMAD* (1.0 ml, 7.92 mmol) in 10 ml toluene was refluxed for 12 h. Then toluene solvent was evaporated to volume ~ 3 ml, and 6 ml of diethyl ether was added to the solution. The next day 0.8 g of the precipitate formed was filtered off and washed with diethyl ether (2×10 ml). The target compound I (0.45 g, 0.83 mmol) was obtained by re–crystallization from ethyl acetate–*DMF* mixture as white solid. Yield 21%. M.p. = 498–500 K. IR, ν/cm^-1^: 1573 (C═C), 1624 (C═O), 1713 (brd.) and 1735 (brd.) (CO_2_*Me*). Mass spectrum, *m*/*z* (I_r_(%)): 544 (21), 543 [*M*^+^] (77), 528 (24), 515 (15), 514 (28), 512 (46), 500 (20), 485 (28), 484 (100), 482 (23), 206 (16), 192 (14), 191 (23), 135 (36), 108 (47), 107 (25), 79 (24), 77 (16), 69 (22), 59 (15). ^1^H NMR (CDCl_3_, 296 K): δ = 6.36 (dd, 1H, H7, ^3^*J* = 5.5, 1.7), 6.28 (d, 1H, H8, ^3^*J* = 5.5), 4.98 (s, 1H, H2'), 4.95 (s, 1H, H4), 4.82 (d, 1H, H6, ^3^*J* = 1.7), 4.65 (brs, 1H, H14), 4.13 (brs, 1H, H10), 3.88 (s, 3H, CO_2_*Me*), 3.74 (s, 3H, CO_2_*Me*), 3.66 (s, 3H, CO_2_*Me*), 3.55 (s, 3H, CO_2_*Me*), 2.83 (m, 2H, H11, H13), 2.13 (d, 1H, H5, ^3^*J* = 6.1), 2.06(d, 1H, H15, ^3^*J* = 6.1), 1.24 (d, 3H, *Me*11, ^3^*J* = 7.8), 1.20 (d, 3H, *Me*13, ^3^*J* = 7.8). ^13^C NMR (CDCl_3_, 296 K): δ = 210.7 (s, C12), 167.7 (s, CO_2_*Me*), 165.4 (s, CO_2_*Me*), 163.0 (s, CO_2_*Me*), 162.2 (s, CO_2_*Me*), 154.9 (s, C1'), 148.3 (brs, C2)^a)^, 144.5 (brs, C3)^a)^, 140.6 (d, C8, *J* = 177.5), 138.6 (d, C7, *J* = 177.5), 90.2 (s, C1)^b)^, 87.4 (s, C9)^b)^, 86.7 (d, C4, *J* = 161.5), 81.0 (d, C2', *J* = 169.5), 80.2 (d, C6, *J* = 165.5), 59.8 (brd, C4, *J* >> 140)^c)^, 55.6 (brd, C10, *J* >> 140)^c)^, 52.8 (q, CO_2_*Me*, *J* = 148.7), 52.5 (q, CO_2_*Me*, *J* = 148.7), 52.0 (q, CO_2_*Me*, *J* = 147.7), 50.8 (q, CO_2_*Me*, *J* = 146.5), 49.5 (d, C5, *J* = 146.0)^d)^, 49.0 (d, C15, *J* = 150.0)^d)^, 43.9 (d, C11, *J* = 137.5)^e)^, 43.5 (d, C13, *J* = 137.5)^e)^, 19.3 (q, *Me*11, *J* = 129.5)^f)^, 19.9 (q, *Me*13, *J* = 129.5)^f)^. The alternative correlation of signals marked by the identical letters is possible.

### Refinement

The hydrogen atoms were placed in calculated positions with C—H = 0.95–1.00Å and refined in the riding model with fixed isotropic displacement parameters: *U*_iso_(H) = 1.5*U*_eq_(C) for CH_3_–groups and *U*_iso_(H) = 1.2*U*_eq_(C) for the other groups.

23 reflections, with experimentally observed *F*^2 ^deviating significantly from the theoretically calculated *F*^2^, were omitted from the refinement. Moreover, 80 reflections were not measured because the angle limits.

### Crystal data

**Table:**

C_27_H_29_NO_11_	*F*(000) = 1144
*M**_r_* = 543.51	*D*_x_ = 1.448 Mg m^−^^3^
Monoclinic, *P*2_1_/*n*	Mo *K*α radiation, λ = 0.71073 Å
Hall symbol: -P 2yn	Cell parameters from 5735 reflections
*a* = 16.1430 (7) Å	θ = 2.4–29.2°
*b* = 9.1365 (4) Å	µ = 0.11 mm^−^^1^
*c* = 16.9658 (7) Å	*T* = 120 K
β = 95.019 (1)°	Prism, colourless
*V* = 2492.70 (18) Å^3^	0.30 × 0.25 × 0.20 mm
*Z* = 4	

### Data collection

**Table:**

Bruker SMART 1K CCD diffractometer	7186 independent reflections
Radiation source: fine–focus sealed tube	5342 reflections with *I* > 2σ(*I*)
graphite	*R*_int_ = 0.041
φ and ω–scans	θ_max_ = 30.0°, θ_min_ = 1.7°
Absorption correction: multi-scan (*SADABS*; Sheldrick, 1998)	*h* = −22→22
*T*_min_ = 0.966, *T*_max_ = 0.975	*k* = −12→12
27638 measured reflections	*l* = −23→23

### Refinement

**Table:**

Refinement on *F*^2^	Primary atom site location: structure-invariant direct methods
Least-squares matrix: full	Secondary atom site location: difference Fourier map
*R*[*F*^2^ > 2σ(*F*^2^)] = 0.054	Hydrogen site location: inferred from neighbouring sites
*wR*(*F*^2^) = 0.151	H-atom parameters constrained
*S* = 1.00	*w* = 1/[σ^2^(*F*_o_^2^) + (0.080*P*)^2^ + 1.3*P*] where *P* = (*F*_o_^2^ + 2*F*_c_^2^)/3
7186 reflections	(Δ/σ)_max_ < 0.001
358 parameters	Δρ_max_ = 0.48 e Å^−^^3^
0 restraints	Δρ_min_ = −0.27 e Å^−^^3^

### Special details

**Table:**

Geometry. All s.u.'s (except the s.u. in the dihedral angle between two l.s. planes) are estimated using the full covariance matrix. The cell s.u.'s are taken into account individually in the estimation of s.u.'s in distances, angles and torsion angles; correlations between s.u.'s in cell parameters are only used when they are defined by crystal symmetry. An approximate (isotropic) treatment of cell s.u.'s is used for estimating s.u.'s involving l.s. planes.
Refinement. Refinement of *F*^2^ against ALL reflections. The weighted *R*–factor *wR* and goodness of fit *S* are based on *F*^2^, conventional *R*–factors *R* are based on *F*, with *F* set to zero for negative *F*^2^. The threshold expression of *F*^2^ > σ(*F*^2^) is used only for calculating *R*–factors(gt) *etc*. and is not relevant to the choice of reflections for refinement. *R*–factors based on *F*^2^ are statistically about twice as large as those based on *F*, and *R*–factors based on ALL data will be even larger.

### Fractional atomic coordinates and isotropic or equivalent isotropic displacement parameters (Å^2^)

**Table:**

	*x*	*y*	*z*	*U*_iso_*/*U*_eq_	
O1	0.66037 (7)	0.73825 (11)	0.16225 (6)	0.0195 (2)	
O2	0.82627 (7)	0.72867 (12)	0.19163 (6)	0.0202 (2)	
O3	0.74127 (8)	0.44442 (13)	0.07291 (7)	0.0293 (3)	
O4	0.48781 (7)	1.10219 (14)	0.06739 (7)	0.0268 (3)	
O5	0.62045 (7)	1.14017 (12)	0.04217 (7)	0.0240 (2)	
O6	0.52775 (8)	0.94821 (14)	0.32897 (7)	0.0287 (3)	
O7	0.49113 (7)	1.11141 (13)	0.23301 (7)	0.0241 (2)	
O8	0.77295 (8)	1.22882 (15)	−0.15191 (8)	0.0336 (3)	
O9	0.64719 (8)	1.16118 (14)	−0.20844 (7)	0.0307 (3)	
O10	0.90731 (7)	1.01714 (13)	−0.09026 (7)	0.0266 (3)	
O11	0.85640 (7)	1.14089 (12)	0.01008 (7)	0.0243 (2)	
N1	0.76190 (8)	0.86475 (14)	0.00228 (7)	0.0180 (2)	
C1	0.67163 (9)	0.86143 (16)	0.11122 (8)	0.0175 (3)	
C2	0.59850 (9)	0.95863 (16)	0.13139 (9)	0.0188 (3)	
C3	0.58971 (9)	0.93231 (17)	0.20836 (9)	0.0198 (3)	
C4	0.65717 (9)	0.82082 (17)	0.23434 (9)	0.0199 (3)	
H4A	0.6466	0.7617	0.2820	0.024*	
C5	0.74072 (9)	0.90776 (17)	0.24083 (9)	0.0191 (3)	
H5A	0.7386	0.9993	0.2729	0.023*	
C6	0.82038 (10)	0.81583 (17)	0.26217 (9)	0.0214 (3)	
H6A	0.8208	0.7589	0.3125	0.026*	
C7	0.89349 (10)	0.91955 (18)	0.25724 (10)	0.0243 (3)	
H7A	0.9270	0.9616	0.3002	0.029*	
C8	0.90099 (9)	0.93947 (17)	0.18049 (9)	0.0220 (3)	
H8A	0.9416	0.9965	0.1571	0.026*	
C9	0.83021 (9)	0.85078 (16)	0.13799 (9)	0.0181 (3)	
C10	0.83343 (9)	0.80270 (17)	0.05110 (8)	0.0186 (3)	
H10A	0.8856	0.8423	0.0315	0.022*	
C11	0.83281 (10)	0.63446 (17)	0.03981 (9)	0.0222 (3)	
H11A	0.8722	0.5907	0.0821	0.027*	
C12	0.74763 (10)	0.56647 (17)	0.04577 (9)	0.0228 (3)	
C13	0.67115 (10)	0.65018 (16)	0.01109 (9)	0.0204 (3)	
H13A	0.6218	0.6154	0.0376	0.024*	
C14	0.68070 (9)	0.81581 (16)	0.02491 (8)	0.0175 (3)	
H14A	0.6365	0.8668	−0.0098	0.021*	
C15	0.75006 (9)	0.93764 (16)	0.15166 (8)	0.0172 (3)	
H15A	0.7536	1.0440	0.1386	0.021*	
C16	0.56052 (9)	1.07353 (17)	0.07847 (9)	0.0196 (3)	
C17	0.59264 (12)	1.25239 (19)	−0.01392 (11)	0.0313 (4)	
H17A	0.6409	1.2969	−0.0358	0.047*	
H17B	0.5621	1.3276	0.0128	0.047*	
H17C	0.5561	1.2088	−0.0568	0.047*	
C18	0.53357 (9)	0.99724 (17)	0.26329 (9)	0.0211 (3)	
C19	0.43121 (11)	1.1740 (2)	0.28212 (10)	0.0302 (4)	
H19A	0.4012	1.2536	0.2534	0.045*	
H19B	0.4603	1.2125	0.3309	0.045*	
H19C	0.3917	1.0983	0.2953	0.045*	
C20	0.86328 (11)	0.5938 (2)	−0.04121 (10)	0.0277 (3)	
H20A	0.8500	0.4911	−0.0532	0.042*	
H20B	0.9236	0.6081	−0.0394	0.042*	
H20C	0.8356	0.6564	−0.0824	0.042*	
C21	0.65541 (11)	0.62021 (18)	−0.07856 (9)	0.0259 (3)	
H21A	0.6602	0.5150	−0.0884	0.039*	
H21B	0.6966	0.6731	−0.1068	0.039*	
H21C	0.5994	0.6535	−0.0974	0.039*	
C22	0.76839 (9)	0.96972 (16)	−0.05400 (8)	0.0184 (3)	
C23	0.70725 (10)	1.01164 (18)	−0.10936 (9)	0.0225 (3)	
H23A	0.6581	0.9544	−0.1172	0.027*	
C24	0.71528 (10)	1.14222 (18)	−0.15703 (9)	0.0233 (3)	
C25	0.64617 (13)	1.2919 (2)	−0.25582 (11)	0.0353 (4)	
H25A	0.5969	1.2913	−0.2940	0.053*	
H25B	0.6964	1.2954	−0.2843	0.053*	
H25C	0.6445	1.3779	−0.2215	0.053*	
C26	0.85240 (9)	1.04562 (17)	−0.04942 (9)	0.0202 (3)	
C27	0.93291 (11)	1.22305 (19)	0.02193 (11)	0.0289 (4)	
H27A	0.9278	1.2971	0.0630	0.043*	
H27B	0.9441	1.2712	−0.0276	0.043*	
H27C	0.9788	1.1565	0.0385	0.043*	

### Atomic displacement parameters (Å^2^)

**Table:**

	*U*^11^	*U*^22^	*U*^33^	*U*^12^	*U*^13^	*U*^23^
O1	0.0250 (5)	0.0177 (5)	0.0160 (5)	−0.0013 (4)	0.0033 (4)	0.0015 (4)
O2	0.0250 (5)	0.0192 (5)	0.0161 (5)	0.0026 (4)	0.0007 (4)	0.0018 (4)
O3	0.0440 (7)	0.0190 (5)	0.0246 (6)	−0.0015 (5)	0.0014 (5)	0.0023 (4)
O4	0.0211 (5)	0.0352 (6)	0.0237 (6)	0.0041 (5)	−0.0015 (4)	−0.0004 (5)
O5	0.0240 (5)	0.0224 (5)	0.0254 (6)	−0.0025 (4)	−0.0001 (4)	0.0054 (4)
O6	0.0299 (6)	0.0364 (7)	0.0205 (5)	0.0039 (5)	0.0063 (4)	0.0049 (5)
O7	0.0258 (6)	0.0265 (6)	0.0209 (5)	0.0056 (4)	0.0066 (4)	0.0001 (4)
O8	0.0315 (7)	0.0329 (7)	0.0358 (7)	−0.0082 (5)	−0.0001 (5)	0.0111 (5)
O9	0.0343 (7)	0.0316 (6)	0.0248 (6)	−0.0023 (5)	−0.0053 (5)	0.0111 (5)
O10	0.0233 (5)	0.0306 (6)	0.0267 (6)	−0.0011 (5)	0.0066 (4)	−0.0008 (5)
O11	0.0248 (6)	0.0238 (6)	0.0243 (6)	−0.0033 (4)	0.0025 (4)	−0.0043 (4)
N1	0.0177 (6)	0.0195 (6)	0.0167 (6)	0.0010 (4)	0.0016 (4)	0.0028 (5)
C1	0.0195 (7)	0.0171 (6)	0.0159 (6)	0.0003 (5)	0.0013 (5)	0.0024 (5)
C2	0.0171 (6)	0.0195 (7)	0.0199 (7)	−0.0014 (5)	0.0019 (5)	0.0002 (5)
C3	0.0193 (7)	0.0207 (7)	0.0192 (7)	−0.0021 (5)	0.0014 (5)	−0.0005 (5)
C4	0.0240 (7)	0.0201 (7)	0.0158 (6)	0.0004 (5)	0.0032 (5)	0.0012 (5)
C5	0.0210 (7)	0.0200 (7)	0.0160 (6)	0.0025 (5)	0.0001 (5)	−0.0002 (5)
C6	0.0243 (7)	0.0222 (7)	0.0172 (7)	0.0037 (6)	−0.0011 (5)	0.0002 (6)
C7	0.0228 (7)	0.0266 (8)	0.0226 (7)	0.0035 (6)	−0.0039 (6)	−0.0035 (6)
C8	0.0186 (7)	0.0219 (7)	0.0249 (7)	0.0001 (5)	−0.0016 (5)	−0.0019 (6)
C9	0.0191 (6)	0.0177 (6)	0.0173 (6)	0.0016 (5)	−0.0001 (5)	0.0004 (5)
C10	0.0184 (6)	0.0200 (7)	0.0174 (7)	0.0021 (5)	0.0012 (5)	0.0000 (5)
C11	0.0281 (8)	0.0190 (7)	0.0191 (7)	0.0040 (6)	0.0008 (6)	−0.0010 (5)
C12	0.0330 (8)	0.0207 (7)	0.0147 (6)	0.0011 (6)	0.0026 (6)	−0.0031 (5)
C13	0.0247 (7)	0.0191 (7)	0.0173 (7)	−0.0035 (5)	0.0021 (5)	−0.0007 (5)
C14	0.0181 (6)	0.0183 (6)	0.0161 (6)	−0.0005 (5)	0.0019 (5)	0.0010 (5)
C15	0.0187 (6)	0.0166 (6)	0.0165 (6)	0.0009 (5)	0.0019 (5)	0.0008 (5)
C16	0.0225 (7)	0.0195 (7)	0.0168 (6)	−0.0002 (5)	0.0009 (5)	−0.0023 (5)
C17	0.0414 (10)	0.0237 (8)	0.0278 (8)	−0.0015 (7)	−0.0029 (7)	0.0080 (7)
C18	0.0184 (7)	0.0243 (7)	0.0206 (7)	−0.0021 (5)	0.0020 (5)	−0.0013 (6)
C19	0.0304 (8)	0.0358 (9)	0.0256 (8)	0.0077 (7)	0.0098 (7)	−0.0033 (7)
C20	0.0303 (8)	0.0291 (8)	0.0240 (8)	0.0062 (7)	0.0042 (6)	−0.0037 (6)
C21	0.0344 (9)	0.0232 (8)	0.0196 (7)	−0.0029 (6)	−0.0011 (6)	−0.0027 (6)
C22	0.0208 (7)	0.0180 (7)	0.0166 (6)	0.0000 (5)	0.0035 (5)	0.0001 (5)
C23	0.0248 (7)	0.0232 (7)	0.0191 (7)	−0.0036 (6)	0.0002 (6)	0.0037 (6)
C24	0.0268 (8)	0.0244 (8)	0.0186 (7)	0.0007 (6)	0.0023 (6)	0.0033 (6)
C25	0.0502 (11)	0.0295 (9)	0.0253 (8)	0.0029 (8)	−0.0018 (8)	0.0103 (7)
C26	0.0214 (7)	0.0194 (7)	0.0196 (7)	−0.0004 (5)	0.0012 (5)	0.0030 (5)
C27	0.0289 (8)	0.0254 (8)	0.0312 (9)	−0.0075 (6)	−0.0030 (7)	−0.0015 (7)

### Geometric parameters (Å, °)

**Table:**

O1—C1	1.4410 (17)	C8—H8A	0.9500
O1—C4	1.4418 (18)	C9—C10	1.543 (2)
O2—C9	1.4447 (18)	C9—C15	1.552 (2)
O2—C6	1.4475 (18)	C10—C11	1.549 (2)
O3—C12	1.214 (2)	C10—H10A	1.0000
O4—C16	1.2013 (19)	C11—C12	1.520 (2)
O5—C16	1.3379 (19)	C11—C20	1.545 (2)
O5—C17	1.4435 (19)	C11—H11A	1.0000
O6—C18	1.2123 (19)	C12—C13	1.526 (2)
O7—C18	1.3265 (19)	C13—C14	1.537 (2)
O7—C19	1.4485 (19)	C13—C21	1.544 (2)
O8—C24	1.219 (2)	C13—H13A	1.0000
O9—C24	1.353 (2)	C14—H14A	1.0000
O9—C25	1.439 (2)	C15—H15A	1.0000
O10—C26	1.2005 (19)	C17—H17A	0.9800
O11—C26	1.3302 (19)	C17—H17B	0.9800
O11—C27	1.4442 (19)	C17—H17C	0.9800
N1—C22	1.3638 (19)	C19—H19A	0.9800
N1—C14	1.4676 (18)	C19—H19B	0.9800
N1—C10	1.4743 (18)	C19—H19C	0.9800
C1—C2	1.540 (2)	C20—H20A	0.9800
C1—C14	1.542 (2)	C20—H20B	0.9800
C1—C15	1.551 (2)	C20—H20C	0.9800
C2—C3	1.347 (2)	C21—H21A	0.9800
C2—C16	1.479 (2)	C21—H21B	0.9800
C3—C18	1.480 (2)	C21—H21C	0.9800
C3—C4	1.528 (2)	C22—C23	1.357 (2)
C4—C5	1.561 (2)	C22—C26	1.519 (2)
C4—H4A	1.0000	C23—C24	1.454 (2)
C5—C6	1.552 (2)	C23—H23A	0.9500
C5—C15	1.558 (2)	C25—H25A	0.9800
C5—H5A	1.0000	C25—H25B	0.9800
C6—C7	1.522 (2)	C25—H25C	0.9800
C6—H6A	1.0000	C27—H27A	0.9800
C7—C8	1.331 (2)	C27—H27B	0.9800
C7—H7A	0.9500	C27—H27C	0.9800
C8—C9	1.529 (2)		
			
C1—O1—C4	96.70 (10)	C12—C13—H13A	108.5
C9—O2—C6	96.06 (11)	C14—C13—H13A	108.5
C16—O5—C17	115.52 (13)	C21—C13—H13A	108.5
C18—O7—C19	115.85 (13)	N1—C14—C13	109.88 (12)
C24—O9—C25	115.81 (14)	N1—C14—C1	109.11 (11)
C26—O11—C27	115.46 (13)	C13—C14—C1	113.24 (12)
C22—N1—C14	121.50 (12)	N1—C14—H14A	108.2
C22—N1—C10	124.01 (12)	C13—C14—H14A	108.2
C14—N1—C10	114.17 (11)	C1—C14—H14A	108.2
O1—C1—C2	100.22 (11)	C1—C15—C9	111.29 (12)
O1—C1—C14	112.76 (12)	C1—C15—C5	101.90 (11)
C2—C1—C14	120.36 (12)	C9—C15—C5	102.02 (11)
O1—C1—C15	103.05 (11)	C1—C15—H15A	113.5
C2—C1—C15	104.77 (11)	C9—C15—H15A	113.5
C14—C1—C15	113.57 (12)	C5—C15—H15A	113.5
C3—C2—C16	129.93 (14)	O4—C16—O5	124.39 (15)
C3—C2—C1	105.09 (13)	O4—C16—C2	126.66 (14)
C16—C2—C1	124.03 (12)	O5—C16—C2	108.93 (12)
C2—C3—C18	131.38 (14)	O5—C17—H17A	109.5
C2—C3—C4	105.41 (13)	O5—C17—H17B	109.5
C18—C3—C4	123.09 (13)	H17A—C17—H17B	109.5
O1—C4—C3	100.46 (11)	O5—C17—H17C	109.5
O1—C4—C5	103.37 (11)	H17A—C17—H17C	109.5
C3—C4—C5	105.71 (12)	H17B—C17—H17C	109.5
O1—C4—H4A	115.2	O6—C18—O7	124.59 (15)
C3—C4—H4A	115.2	O6—C18—C3	122.12 (15)
C5—C4—H4A	115.2	O7—C18—C3	113.29 (13)
C6—C5—C15	99.94 (11)	O7—C19—H19A	109.5
C6—C5—C4	115.74 (13)	O7—C19—H19B	109.5
C15—C5—C4	100.20 (11)	H19A—C19—H19B	109.5
C6—C5—H5A	113.1	O7—C19—H19C	109.5
C15—C5—H5A	113.1	H19A—C19—H19C	109.5
C4—C5—H5A	113.1	H19B—C19—H19C	109.5
O2—C6—C7	100.94 (12)	C11—C20—H20A	109.5
O2—C6—C5	102.59 (11)	C11—C20—H20B	109.5
C7—C6—C5	106.32 (13)	H20A—C20—H20B	109.5
O2—C6—H6A	115.1	C11—C20—H20C	109.5
C7—C6—H6A	115.1	H20A—C20—H20C	109.5
C5—C6—H6A	115.1	H20B—C20—H20C	109.5
C8—C7—C6	106.04 (14)	C13—C21—H21A	109.5
C8—C7—H7A	127.0	C13—C21—H21B	109.5
C6—C7—H7A	127.0	H21A—C21—H21B	109.5
C7—C8—C9	105.12 (14)	C13—C21—H21C	109.5
C7—C8—H8A	127.4	H21A—C21—H21C	109.5
C9—C8—H8A	127.4	H21B—C21—H21C	109.5
O2—C9—C8	100.63 (11)	C23—C22—N1	125.97 (14)
O2—C9—C10	112.88 (12)	C23—C22—C26	119.91 (13)
C8—C9—C10	121.00 (13)	N1—C22—C26	114.09 (12)
O2—C9—C15	102.57 (11)	C22—C23—C24	121.47 (14)
C8—C9—C15	104.88 (12)	C22—C23—H23A	119.3
C10—C9—C15	112.73 (11)	C24—C23—H23A	119.3
N1—C10—C9	109.69 (12)	O8—C24—O9	122.35 (15)
N1—C10—C11	108.41 (12)	O8—C24—C23	126.71 (15)
C9—C10—C11	113.59 (12)	O9—C24—C23	110.90 (14)
N1—C10—H10A	108.3	O9—C25—H25A	109.5
C9—C10—H10A	108.3	O9—C25—H25B	109.5
C11—C10—H10A	108.3	H25A—C25—H25B	109.5
C12—C11—C20	108.73 (13)	O9—C25—H25C	109.5
C12—C11—C10	113.16 (13)	H25A—C25—H25C	109.5
C20—C11—C10	110.46 (13)	H25B—C25—H25C	109.5
C12—C11—H11A	108.1	O10—C26—O11	126.20 (14)
C20—C11—H11A	108.1	O10—C26—C22	124.93 (14)
C10—C11—H11A	108.1	O11—C26—C22	108.74 (12)
O3—C12—C11	120.50 (15)	O11—C27—H27A	109.5
O3—C12—C13	120.94 (15)	O11—C27—H27B	109.5
C11—C12—C13	118.40 (13)	H27A—C27—H27B	109.5
C12—C13—C14	111.57 (12)	O11—C27—H27C	109.5
C12—C13—C21	110.41 (13)	H27A—C27—H27C	109.5
C14—C13—C21	109.30 (12)	H27B—C27—H27C	109.5
			
C4—O1—C1—C2	−52.41 (12)	C11—C12—C13—C21	83.94 (16)
C4—O1—C1—C14	178.34 (12)	C22—N1—C14—C13	121.65 (14)
C4—O1—C1—C15	55.50 (12)	C10—N1—C14—C13	−64.54 (15)
O1—C1—C2—C3	32.96 (14)	C22—N1—C14—C1	−113.65 (14)
C14—C1—C2—C3	157.09 (13)	C10—N1—C14—C1	60.16 (15)
C15—C1—C2—C3	−73.59 (14)	C12—C13—C14—N1	48.24 (16)
O1—C1—C2—C16	−157.27 (13)	C21—C13—C14—N1	−74.16 (15)
C14—C1—C2—C16	−33.1 (2)	C12—C13—C14—C1	−74.04 (16)
C15—C1—C2—C16	96.17 (15)	C21—C13—C14—C1	163.56 (13)
C16—C2—C3—C18	7.4 (3)	O1—C1—C14—N1	−119.65 (13)
C1—C2—C3—C18	176.34 (15)	C2—C1—C14—N1	122.40 (13)
C16—C2—C3—C4	−168.69 (15)	C15—C1—C14—N1	−2.90 (16)
C1—C2—C3—C4	0.24 (15)	O1—C1—C14—C13	3.06 (17)
C1—O1—C4—C3	52.76 (12)	C2—C1—C14—C13	−114.90 (14)
C1—O1—C4—C5	−56.33 (12)	C15—C1—C14—C13	119.81 (13)
C2—C3—C4—O1	−33.38 (15)	O1—C1—C15—C9	74.61 (13)
C18—C3—C4—O1	150.11 (13)	C2—C1—C15—C9	179.06 (11)
C2—C3—C4—C5	73.85 (15)	C14—C1—C15—C9	−47.69 (16)
C18—C3—C4—C5	−102.66 (15)	O1—C1—C15—C5	−33.49 (13)
O1—C4—C5—C6	−71.63 (15)	C2—C1—C15—C5	70.96 (13)
C3—C4—C5—C6	−176.75 (12)	C14—C1—C15—C5	−155.79 (12)
O1—C4—C5—C15	34.72 (13)	O2—C9—C15—C1	−75.60 (13)
C3—C4—C5—C15	−70.39 (13)	C8—C9—C15—C1	179.64 (12)
C9—O2—C6—C7	−51.13 (12)	C10—C9—C15—C1	46.09 (16)
C9—O2—C6—C5	58.53 (12)	O2—C9—C15—C5	32.42 (13)
C15—C5—C6—O2	−37.45 (14)	C8—C9—C15—C5	−72.34 (14)
C4—C5—C6—O2	69.06 (15)	C10—C9—C15—C5	154.11 (12)
C15—C5—C6—C7	68.10 (14)	C6—C5—C15—C1	117.98 (12)
C4—C5—C6—C7	174.61 (12)	C4—C5—C15—C1	−0.67 (13)
O2—C6—C7—C8	31.52 (16)	C6—C5—C15—C9	2.87 (14)
C5—C6—C7—C8	−75.21 (16)	C4—C5—C15—C9	−115.78 (12)
C6—C7—C8—C9	1.65 (16)	C17—O5—C16—O4	−0.5 (2)
C6—O2—C9—C8	52.09 (12)	C17—O5—C16—C2	177.92 (13)
C6—O2—C9—C10	−177.52 (12)	C3—C2—C16—O4	−52.2 (3)
C6—O2—C9—C15	−55.94 (12)	C1—C2—C16—O4	140.73 (16)
C7—C8—C9—O2	−34.40 (15)	C3—C2—C16—O5	129.43 (17)
C7—C8—C9—C10	−159.45 (14)	C1—C2—C16—O5	−37.64 (19)
C7—C8—C9—C15	71.79 (15)	C19—O7—C18—O6	−3.3 (2)
C22—N1—C10—C9	111.90 (15)	C19—O7—C18—C3	176.24 (13)
C14—N1—C10—C9	−61.74 (16)	C2—C3—C18—O6	171.50 (16)
C22—N1—C10—C11	−123.55 (15)	C4—C3—C18—O6	−13.0 (2)
C14—N1—C10—C11	62.81 (15)	C2—C3—C18—O7	−8.1 (2)
O2—C9—C10—N1	120.82 (13)	C4—C3—C18—O7	167.45 (13)
C8—C9—C10—N1	−120.03 (14)	C14—N1—C22—C23	−19.1 (2)
C15—C9—C10—N1	5.17 (17)	C10—N1—C22—C23	167.72 (15)
O2—C9—C10—C11	−0.66 (17)	C14—N1—C22—C26	158.92 (13)
C8—C9—C10—C11	118.48 (15)	C10—N1—C22—C26	−14.3 (2)
C15—C9—C10—C11	−116.31 (14)	N1—C22—C23—C24	168.41 (14)
N1—C10—C11—C12	−46.70 (16)	C26—C22—C23—C24	−9.5 (2)
C9—C10—C11—C12	75.50 (16)	C25—O9—C24—O8	−1.2 (2)
N1—C10—C11—C20	75.47 (15)	C25—O9—C24—C23	176.67 (15)
C9—C10—C11—C20	−162.33 (13)	C22—C23—C24—O8	−2.7 (3)
C20—C11—C12—O3	89.83 (17)	C22—C23—C24—O9	179.48 (15)
C10—C11—C12—O3	−147.04 (14)	C27—O11—C26—O10	4.3 (2)
C20—C11—C12—C13	−85.50 (16)	C27—O11—C26—C22	−179.67 (13)
C10—C11—C12—C13	37.63 (18)	C23—C22—C26—O10	−82.3 (2)
O3—C12—C13—C14	146.87 (14)	N1—C22—C26—O10	99.52 (18)
C11—C12—C13—C14	−37.82 (18)	C23—C22—C26—O11	101.56 (16)
O3—C12—C13—C21	−91.37 (17)	N1—C22—C26—O11	−76.58 (16)

### Hydrogen-bond geometry (Å, °)

**Table:**

*D*—H···*A*	*D*—H	H···*A*	*D*···*A*	*D*—H···*A*
C19—H19C···O8^i^	0.98	2.70	3.011 (2)	99

Symmetry codes: (i) *x*−1/2, −*y*+5/2, *z*+1/2.
